# Emerging Concepts and Novel Strategies in Radiation Therapy for Laryngeal Cancer Management

**DOI:** 10.3390/cancers12061651

**Published:** 2020-06-22

**Authors:** Mauricio E. Gamez, Adriana Blakaj, Wesley Zoller, Marcelo Bonomi, Dukagjin M. Blakaj

**Affiliations:** 1 Division of Radiation Oncology, The Ohio State University Wexner Medical Center, Columbus, OH 43210, USA; Wesley.Zoller@osumc.edu (W.Z.); Dukagjin.Blakaj@osumc.edu (D.M.B.); 2 Department of Therapeutic Radiology, Yale School of Medicine, 35 Park St., New Haven, CT 06519, USA; Adriana.Blakaj@yale.edu; 3 Department of Internal Medicine, Division of Medical Oncology, The Ohio State University Wexner Medical Center, 320 West 10th Avenue, Columbus, OH 43210, USA; Marcelo.Bonomi@osumc.edu

**Keywords:** laryngeal cancer, radiotherapy, IMRT, IGRT, SABR, de-escalation therapy

## Abstract

Laryngeal squamous cell carcinoma is the second most common head and neck cancer. Its pathogenesis is strongly associated with smoking. The management of this disease is challenging and mandates multidisciplinary care. Currently, accepted treatment modalities include surgery, radiation therapy, and chemotherapy—all focused on improving survival while preserving organ function. Despite changes in smoking patterns resulting in a declining incidence of laryngeal cancer, the overall outcomes for this disease have not improved in the recent past, likely due to changes in treatment patterns and treatment-related toxicities. Here, we review emerging concepts and novel strategies in the use of radiation therapy in the management of laryngeal squamous cell carcinoma that could improve the relationship between tumor control and normal tissue damage (therapeutic ratio).

## 1. Introduction

Laryngeal cancer is the second most common head and neck cancer, and it represents about a fifth of the total head and neck cancer diagnoses [1]. The median age at diagnosis is 65 years, with men more commonly affected than women, and with a higher incidence and mortality among black individuals [2,3]. The Global Cancer Observatory reported a total of 177,000 estimated new cases of laryngeal cancer and 94,000 deaths in 2018 [4]. In the United States, there were 12,370 estimated new cases (9820 in men, and 2550 in women) and 3750 deaths (3000 men and 750 women) from laryngeal cancer this year [5]. The vast majority (>95%) are squamous cell carcinoma (SCC), and smoking is the main risk factor [6].

The larynx is an organ of the upper aerodigestive tract involved in three vital functions: occlusion/protection of the airway during swallowing, phonation, and breathing. In consequence, the management of SCC of the larynx is challenging, as both the cancer itself and its treatment may significantly impact function and quality of life. For purposes of cancer staging, the larynx is divided into three anatomic regions: the supraglottis, glottis, and subglottis. The incidence of larynx cancer in the glottis represents the majority of cases (69%), whereas the cancer originating in the supraglottis (30%) and subglottis (1%) is less common [7,8,9,10]. 

Currently accepted treatment modalities in the management of laryngeal cancer include surgery, radiation therapy (RT), and chemotherapy. Decision-making is primarily based on tumor location, histology, staging, baseline function, and in some cases, patient preference.

The aim of the manuscript is to review the emerging concepts and newer strategies with the use of RT in the management of laryngeal cancer, focusing on specific clinical scenarios, with the goal of improving the delicate balance between tumor control and damage to surrounding normal tissue, known as the therapeutic ratio. It is important to note that currently, none of the therapeutic approaches discussed below are standard care or routinely used in clinical practice, but represent novel treatment strategies in larynx cancer with the potential for improved outcomes.

## 2. Early-Stage Glottic Cancer (I–II)

At diagnosis, most glottic cancers are confined to the true vocal cords, usually one cord, with most occurring at the medial and superior aspects of the anterior two-thirds of the vocal folds [11,12]. Persisting hoarseness is typically the presenting symptom. Tumors limited to one vocal cord (T1a) or involving both (T1b) cords can be successfully treated with single modality therapy, either surgery (endoscopic techniques) or RT alone, resulting in excellent oncologic outcomes with 5year local control (LC) rates at around 90–95%. With respect to functional outcomes, specifically voice quality preservation rates, there has not been a head-to-head comparison between both treatment modalities; however, small, single-institution studies suggest equal or even slightly better voice quality after RT [13,14,15,16]. A currently accruing clinical trial (NCT04057209) will evaluate voice quality after transoral CO_2_laser surgery vs. RT for cTis (carcinoma in situ) or cT1a of the glottic larynx.

RT has been a key component in the management of laryngeal cancer for the last century [17,18]. Historically, the radiation treatment fields employed for Tis and T1 glottic cancers have consisted of two opposed lateral fields (small laryngeal fields), 5 × 5 cm in size, defined by anatomic landmarks, also called “conventional radiation” [19,20]. The optimal radiation dose, fraction size, and overall treatment time for local control in early stage glottic cancers with conventional radiation was evaluated in a Japanese prospective randomized trial [21]. The use of a slightly hypofractionated radiation regimen (2.25 Gy per fraction) applied over a shorter overall treatment time was superior to conventional fractionation (2 Gy per fraction), with respect to local control and without increasing toxicity. Currently accepted RT doses for the management of Tis and T1 glottic larynx cancer are 60.75 Gy and 63 Gy at 2.25 Gy per fraction, respectively.

A limitation of conventional radiation is the total cumulative dose that the surrounding normal tissues will receive, particularly the pharyngeal constrictor muscles, submandibular and thyroid glands, and carotid arteries, raising the concern for increased risk of late complications, such as dysphagia, aspiration, xerostomia, hypothyroidism, and cerebrovascular events [22,23], which could negatively impact long-term outcomes. Therefore, there is a growing interest in the use of modern RT modalities like intensity-modulated radiation therapy (IMRT), stereotactic ablative radiation therapy (SABR), and charged particle therapy, with the attempt to decrease treatment-related toxicities while maintaining, if not improving, outcomes.

### 2.1. Carotid-Sparing IMRT

**Figure 1:**
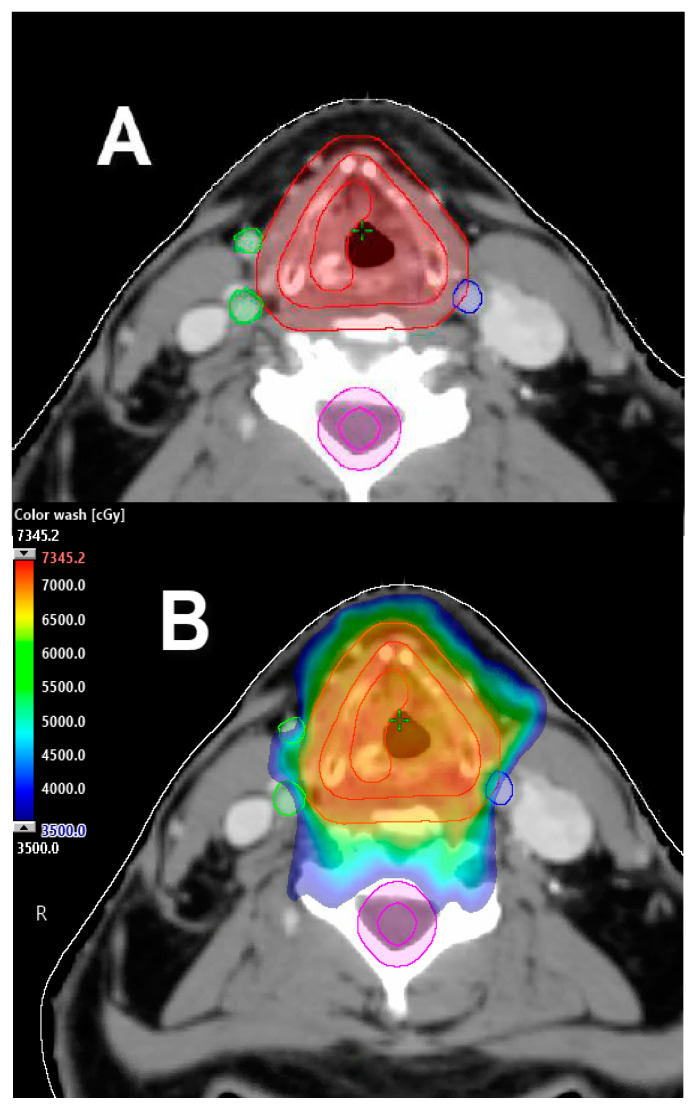
Exemplification of carotid-sparing intensity-modulated radiation therapy (IMRT) plan. (**A**) Carotid arteries delineated using contrast-enhanced computed tomography (CT) simulation to be avoided during planning. (**B**) Carotid-sparing volumetric modulated arc therapy (VMAT) approach for T1a right-sided true vocal cord carcinoma; 63 Gy prescription delivered in 28 fractions. Dose color wash distribution.

The MD Anderson Cancer Center group reported a large retrospective analysis comparing conventional radiation vs. IMRT for the treatment of T1 glottic cancer. The IMRT technique in this study limited the dose to the carotid arteries to as low as reasonably possible given no established dose thresholds for carotid artery toxicity. After a median follow-up of 68 months, there were no significant differences in local or locoregional control and overall survival (OS) between treatment techniques. Interestingly, four patients (3%) had post-radiation cerebrovascular events in the conventional radiation group vs. none in the cohort treated with IMRT. This was a single-institution study with relatively small numbers; however, this analysis may suggest that modern RT techniques such as IMRT can potentially be associated with a decreased risk of post-radiation cerebrovascular events compared to conventional radiation [24] (Figure 1).

Similarly, a National Cancer Database (NCDB) Analysis [25] compared the outcomes of three-dimensional (3D)-conformal radiotherapy (n = 2696) and IMRT (n = 1623) for the treatment of early-stage glottic cancer. No difference in OS was found between groups, with a five-year OS of 72%. In a subset analysis, a survival benefit was observed in the group of patients that received hypofractionated RT (2.25 Gy per fraction) compared to those treated with conventional fractionation (2 Gy per fraction): 76% vs. 70%, respectively. Due to limitations in the analysis of the NCDB, no conclusions could be made with respect to toxicities between the two treatment modalities.

Although current retrospective data with the use of IMRT for early-stage glottic cancer seem to support its efficacy and safety, to date there is no prospective data to show better outcomes or decreased toxicity with IMRT. The radiation oncologist should place careful attention on the selection of treatment volume margins to take into account larynx motion due to respiration and deglutition during treatment and on daily setup, with the use of image-guided radiation therapy (IGRT) to avoid potential marginal misses with the use of this technique.

### 2.2. Single Vocal Cord Irradiation

Traditionally, since the implementation of RT in the management of early stage glottic cancers, the entire anatomic larynx/hypopharynx complex is included and treated in the radiation fields, which is likely explained by the historical diagnostic and technologic limitations in the delivery of RT. We know that at diagnosis, most cases will have disease only in one vocal cord. Furthermore, endoscopic surgical resection only addresses the involved vocal cord. Therefore, the question of whether with newer and better technology the treatment of a single vocal cord with RT is feasible, safe, and could further improve the toxicity profile, has yet to be answered. 

The group from Erasmus Medical Center, Rotterdam, Netherlands, explored in a stepwise manner the feasibility of single vocal cord irradiation for T1a SCC of the glottic larynx. First, using four-dimensional (4D) computed tomography (CT) data, they tested vocal cord mobility and daily reproducibility of the approach, showing a small intra-fraction motion of the vocal cords [26]. Second, they evaluated the adequacy of the daily positioning/set-up with cone-beam CT imaging [27]. Then, they analyzed the dosimetric and potential clinical advantages of the use of IMRT for single vocal cord irradiation [28]. Using this methodology, they evaluated ten patients previously treated with conventional RT. Single vocal cord IMRT plans were created, and estimated local control rates were similar to conventional radiation, but with a better voice handicap index (VHI) [29]. Lately, they reported their initial clinical results for thirty patients treated with single vocal cord irradiation. Patients were treated with IMRT with a hypofractionated scheme, to a total dose of 58.08 Gy in 16 fractions of 3.63 Gy, five times per week. After a median follow-up of 30 months, no single local failure was documented, and two-year OS was 90%. Only one patient developed grade 2 laryngeal edema, which was treated successfully with steroids. A slight and temporary deterioration of VHI scores was noted at the end of treatment, yet started to improve four weeks after and returned back to normal levels around six weeks following RT completion [30].

Currently, two randomized trials are evaluating this question: a randomized study of vocal-cord only vs. complete laryngeal radiotherapy for early glottic cancer (VOCAL; NCT03759431) and a randomized trial evaluating voice quality after transoral CO_2_-laser surgery vs. single vocal cord irradiation for larynx cancer (VoiceS; NCT04057209).

**Figure 2:**
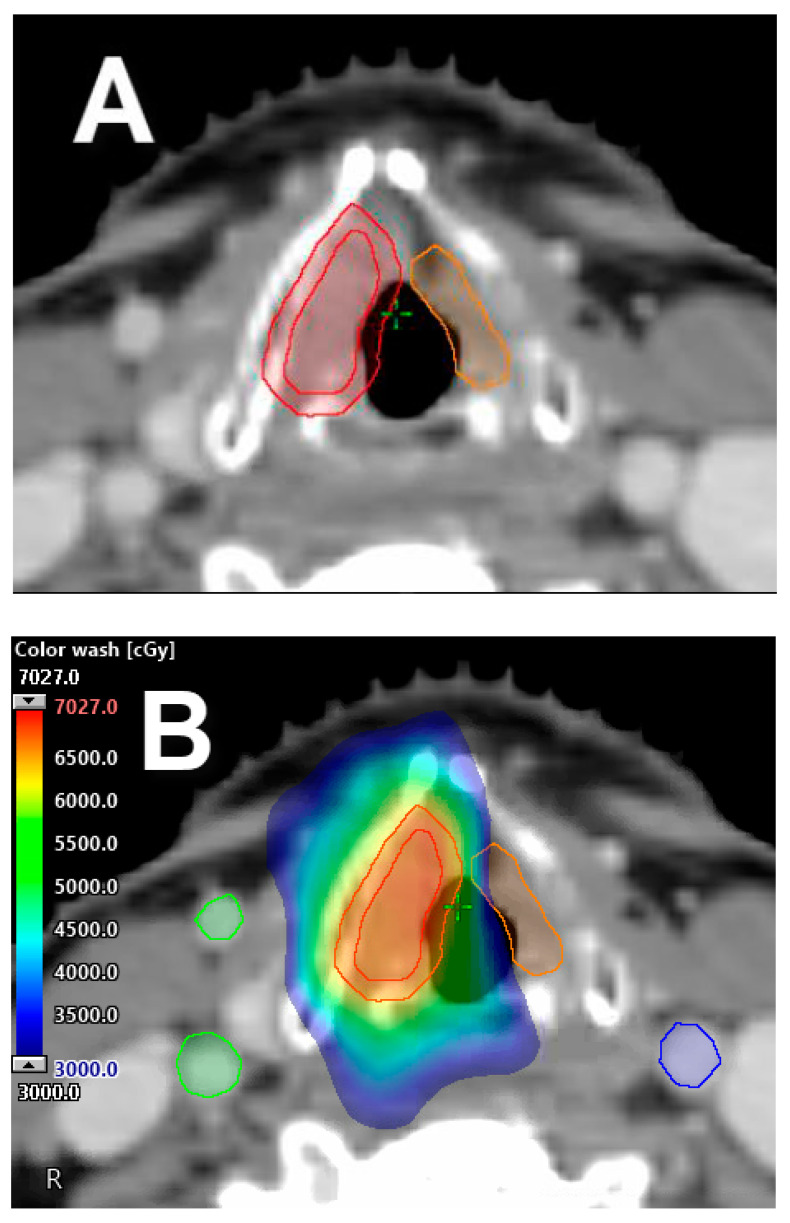
Single vocal cord irradiation. (**A**) The affected cord is delineated as the clinical target volume (CTV), with 5 mm expansion in all directions to generate the planning target volume (PTV) (red). (**B**) Single cord irradiation planned with IMRT treatment technique for T1a, right-sided, true vocal cord carcinoma; a 63 Gy prescription was delivered in 28 fractions, with contralateral vocal cord mean dose kept under 30 Gy. Dose color wash distribution.

An interesting clinical thought is if the low-dose radiation bath delivered to the contralateral, uninvolved vocal cord is sufficient to treat potential microscopic disease without resulting in increased rates of contralateral cord failure and the need for further treatment (Figure 2). If this concept is clinically proven, the next logical step and ultimate way to spare the contralateral vocal cord will be to use charged particle radiation therapy (i.e., proton therapy) for the treatment of T1a glottic cancer. With this technique, however, careful attention and evaluation is imperative, particularly in tumors located at the anterior third of the vocal cord that can extend/involve the anterior commissure. To date, the feasibility and safeness of this approach remains under investigation and is unknown.

### 2.3. Moderate–Extreme Hypofractionation

**Figure 3:**
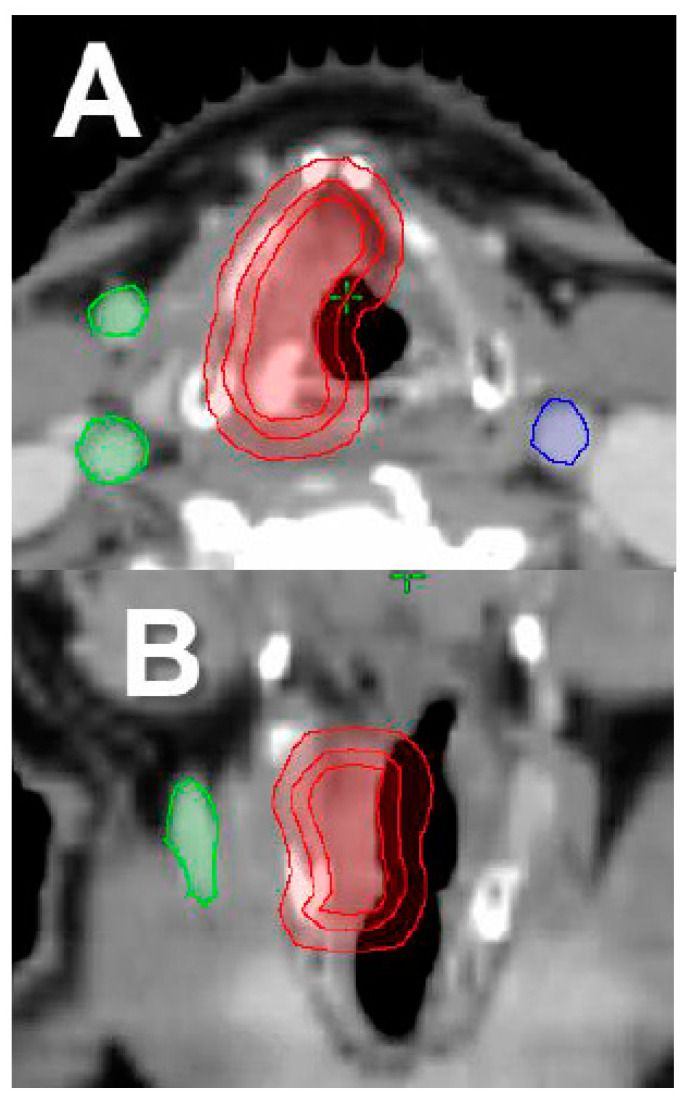

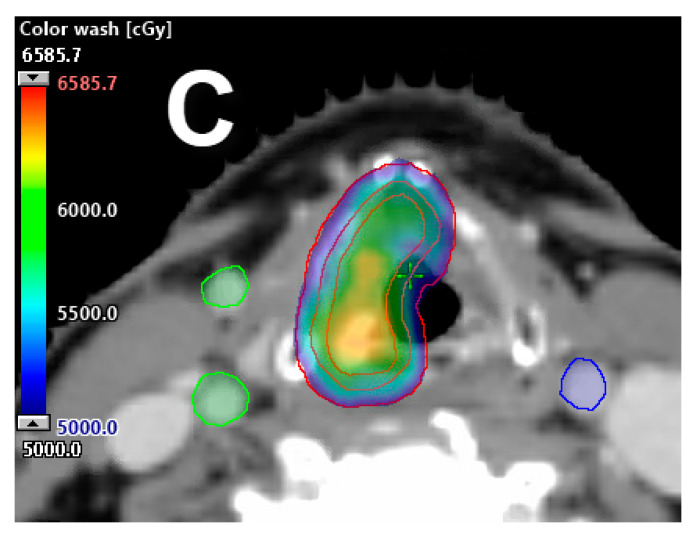
Exemplification of Stereotactic ablative radiation therapy (SABR) treatment plan. (**A**) Axial cross-section, target delineated by expanding the gross tumor volume (GTV) 3 mm uniformly to establish CTV, with uniform 5 mm expansion from the CTV to generate PTV. (**B**) Coronal-view of GTV, CTV, and PTV (red). (**C**) SABR VMAT approach to a single cord, with a prescription dose of 50 Gy in 15 daily fractions. Dose color wash distribution.

Another area of recent interest is in the delivery of stereotactic ablative radiation therapy (SABR) (Figure 3). The University of Texas Southwestern (UTSW) group performed a phase I dose escalation study of SABR in early-stage glottic cancer (Tis–T2 disease). All patients were simulated using 4D-CT, and all radiation therapy was delivered using the Cybernkife Robotic Radiosurgery System (Accuray Inc, Sunnyvale, CA, USA). An internal gross tumor volume (IGTV) was created to encompass gross disease throughout the respiratory cycle. Then, a clinical target volume (CTV) was created by expanding the IGTV by 2 mm, and the planning target volume (PTV) was formed by an expansion of 3 mm on the CTV. A total of 29 patients were enrolled in the trial. Three dose levels—50 Gy in 15 daily fractions (n = 4), 45 Gy in 10 thrice-weekly fractions (n = 13), and 42.5 Gy in five twice-weekly fractions (n = 12)—were evaluated. After a median follow-up of 39 months, two patients developed dose-limiting toxicity, a patient treated with 45 Gy in 10 fractions developed grade 4 laryngeal edema and grade 3 dysphagia, and the other patient treated with 42.5 Gy in five fractions developed grade 3 laryngeal necrosis. Both cases occurred on actively smoking patients with large treatment volumes (PTV ≥ 17 cc). Two recurrences in the group of patients treated with 50 Gy in 15 fractions, and three in the group treated with 45 Gy in 10 fractions, were documented. The VHI results demonstrated excellent long-term voice outcomes. Based on this preliminary data, they concluded that SABR delivered in five fractions can be efficacious and tolerable in this selected group of patients [31]. 

Interestingly, Kang et al. [32] reported the results of their phase I dose escalation trial using SABR in early-stage glottic cancer. A total of thirteen patients were enrolled. Volumetric modulated arc therapy (VMAT) with simultaneous integrated boost (SIB) was used. CTV1 was defined as the gross tumor volume (GTV), and CTV2 included the remaining larynx from the thyroid notch to the inferior portion of the cricoid cartilage. The PTV was formed by adding a 3 mm margin in all directions to the CTV. Seven patients received 59.5 Gy in 17 fractions to PTV1, and 47.6 Gy in 17 fractions to PTV2, 5 days per week. The last six patients received 55 Gy in 11 fractions to PTV1, and 40.7 Gy in 11 fractions to PTV2, every other day, twice per week. The median follow-up for the first treatment group was 37 months, and for the second group was 14.5 months. In the former treatment group, no chronic toxicity was observed, and in the latter treatment group, two (33.3%) of six patients experienced late dose-limiting toxicities (grade 3 laryngeal inflammation). The trial was terminated early due to unacceptably high risk of toxicity.

The discrepancies in the results between the two protocols could be explained by their differences in target volume definitions, larynx motion (use of 4D CT for treatment planning), treatment volume sizes, delivered radiation doses, use of IGRT for daily setup, and clinical factors, such as current smoking status. Furthermore, different institutions advocate for not adding a margin from GTV to CTV (i.e., GTV = CTV), to reduce total treatment volume and normal tissue complications [30,33]. However, special caution should be taken in the radiation treatment planning and setup of these cases, to avoid potential marginal misses. To date, the use of SABR for the treatment of early-stage glottic cancer remains in an experimental phase.

To further evaluate this question of SABR in early-stage glottic cancer, clinical trial NCT03548285 is currently accruing. Patients are stratified by low- and moderate-risk categories based on planning target volume (PTV) and smoking status. Patients in the low-risk category will have PTV < 10 cc, no reported smoking within 1 month from registration, and RT will be delivered twice per week for a total dose of 42.5 Gy in five fractions. Patients in the moderate-risk category will encompass those with PTV ≥ 10 cc, smoking within 1 month from registration (no more than 1 pack per day), and RT for these patients will be delivered daily for a total dose of 58.08 Gy in 16 fractions.

### 2.4. Partial Laryngeal IMRT

T2 laryngeal tumors span a range of different clinical scenarios, from tumors extending to the supraglottic and subglottic larynx (old T2a stage), to tumors with impaired vocal cord mobility (old T2b stage). Therefore, controversy exists with regard to a better approach and management of these tumors. Some groups advocate using 6 × 6 cm opposed lateral fields depending on the degree of supraglottic or subglottic extension. Other groups recommend treating/adding lymph node levels II and III, based on the risk of lymphatic involvement. Radiation Therapy Oncology Group (RTOG) 95-12 [34] analyzed 239 patients with T2N0 squamous cell carcinoma of the glottic larynx treated with definitive RT, and randomized them to standard fractionation 70 Gy in 35 fractions vs. hyperfractionation of 79.2 Gy in 66 fractions at 1.2 Gy bid. Patients in the standard arm were treated with two-dimensional RT using 2 or 3 co-planar portals (6 × 6 cm), allowing field reductions at 50 Gy to reduce the dose to the arytenoids. Patients that received hyperfractionation could have field reductions at 60 Gy. Regional lymph nodes were not intentionally treated, except for a portion of levels II and III that were in the treatment fields. The trial was powered to detect 15% absolute difference in local control at 5 years. After a median follow-up of 7.9 years, the five-year local control was higher for hyperfractiontion (78%) vs. standard fractionation (70%), but it did not meet the primary endpoint. No differences in disease-free survival (DFS) and OS were noted. Higher rates of acute toxicity (skin, mucosal, larynx) were associated with hyperfractionation.

Princess Margaret Cancer Centre reported their experience with the treatment of partial laryngeal IMRT for T2N0 glottic cancer. In their series, the GTV was delineated based on endoscopic and radiographic findings, and expanded 5 mm to create the high-dose CTV. Another 5 mm were added to form the low-dose CTV. The PTV was a systematic expansion of 5 mm radially and 10 mm superiorly and inferiorly. Patients were treated either with hypofractionated IMRT 60 Gy in 25 fractions, or with accelerated IMRT 66–70 Gy/33–35 fractions over a period of 5.5–6.0 weeks, using image guidance matched to cervical vertebrae or laryngeal soft tissue. The three-year LC was significantly higher for accelerated IMRT/IGRT–larynx (89%) vs. hypofractionated IMRT/IGRT–larynx (80%) vs. hypofractionated IMRT/IGRT–bone (70%) [35].

The five-year LC rates of non-favorable T2 (bulky, impaired cord mobility; old T2b stage) glottic lesions is around 70% [36,37,38]. Their management is challenging and an area of research interest. Different treatment intensification strategies have been advocated, ranging from accelerated fractionation, to hyperfractionation, to concurrent chemoradiatotherapy aiming to improve outcomes [39,40,41,42].

**Figure 4:**
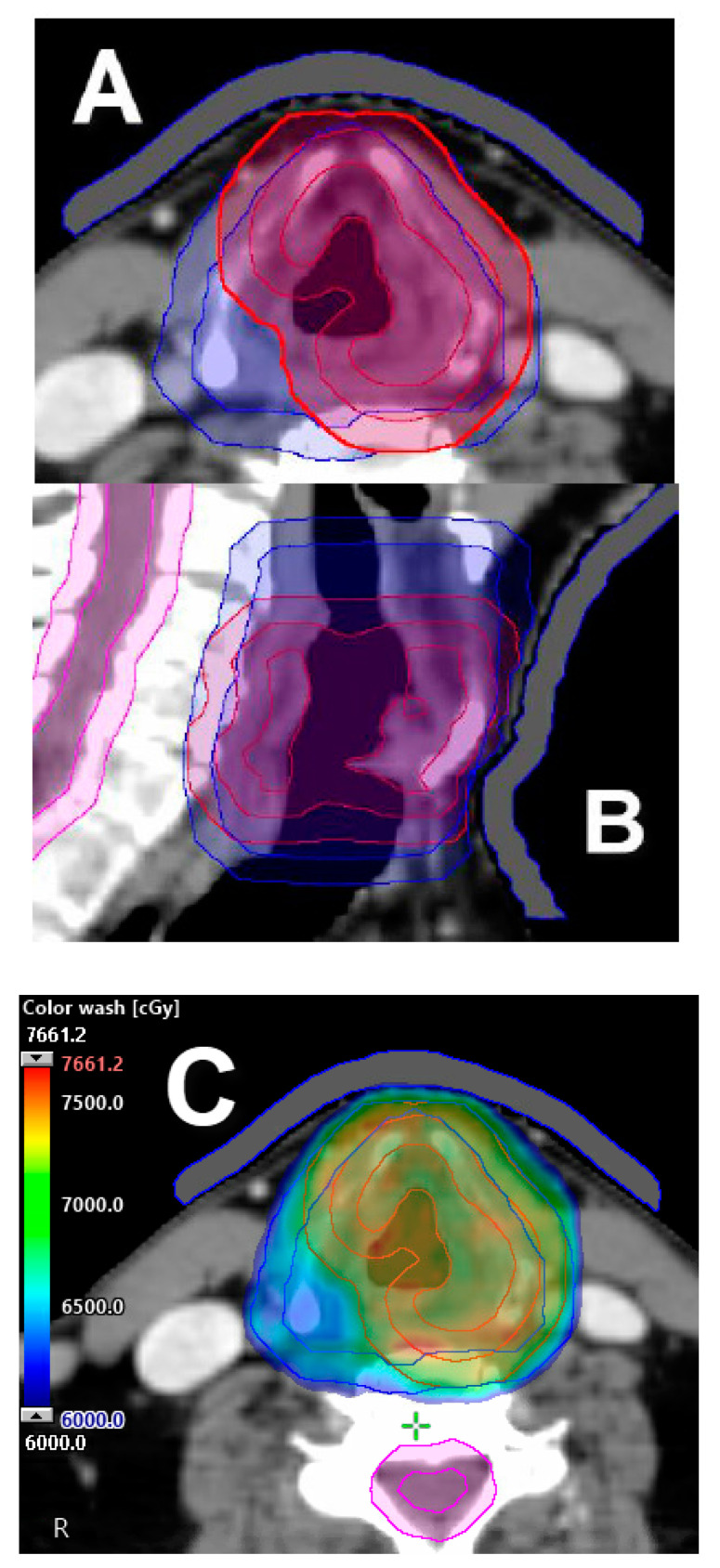
Partial laryngeal IMRT. (**A**) Axial view of target volume delineation for T2N0 glottic larynx (impaired cord mobility) squamous cell carcinoma: primary GTV (red), 5 mm uniform expansion to CTV (red), and 5 mm uniform expansion from CTV to render primary PTV (red). Intermediate CTV (blue) consists of the larynx (CTV) superior to the hyoid, with uniform 5 mm expansion to render intermediate PTV. (**B**) Sagittal view of both high and intermediate target volumes. (**C**) Partial IMRT approach with 5 mm bolus utilizing a simultaneous integrated boost method: 70 Gy in 35 fractions to primary PTV (red), with 56 Gy to intermediate risk PTV (blue). Dose color wash distribution.

The currently recommended standard-of-care RT doses and fractionations for the treatment of favorable T2 glottic lesions (non-bulky, normal cord mobility; old T2a stage) are 65.25 Gy at 2.25 Gy per fraction or 70 Gy at 2 Gy per fraction (Figure 4). For non-favorable T2 (borderline T3) glottic lesions, therapy intensification with hyperfractionation [34,36,37] or concurrent chemoradiation may be considered [39,40,41,42].

## 3. Locally Advanced Stage (III–IV)

Locally advanced disease represents about 60% of all laryngeal cancers. Typically, the management of these tumors will require multimodality therapy. Accepted treatment modalities include surgery followed by adjuvant radiation and chemotherapy, based on high-risk pathologic features, organ-preservation chemoradiation, and induction chemotherapy, followed by definitive chemoradiation. An area of particular interest is how to accurately select the patient who is suitable for organ preservation treatment. The optimal treatment strategy is contingent on multiple factors, including initial tumor extent/stage, baseline/pre-treatment organ function, specific treatment goals, patient preferences, oncologist experience, cancer center volume, and patient compliance for close monitoring and detection of early recurrences [43,44,45,46,47]. In order to choose the appropriate treatment course for an individual case, is imperative to consistently have a multidisciplinary approach [48]. Here, we present different strategies that can help to better select, further tailor, and individualize therapy with the use of radiation.

### 3.1. Tumor Volume

The importance of the pretreatment tumor volume of laryngeal tumors treated with RT and its relationship with outcomes has been demonstrated in multiple studies [49,50,51]. The University of Florida evaluated primary tumor volume as a useful measure to better select laryngeal cancer patients for organ preservation chemoradiation, using pretreatment computed tomography measurements [52]. A pretreatment tumor volume ≤12 cc was a predictor for local control and larynx function preservation after chemoradiation, particularly in the setting of a supraglottic tumor. In addition, the University of Michigan analyzed the value of anatomic volumes in untreated laryngeal cancer patients and demonstrated the prognostic significance of the size of the primary tumor volume, composite nodal volume, and composite total tumor volume [53].

Other groups have further evaluated this concept, using alternative diagnostic imaging modalities, such as magnetic resonance Imaging (MRI), positron emission tomography (PET)/CT, and single-photon emission computed tomography (SPECT)/CT, and analyzing endpoints, such as metabolic tumor volume (MTV), maximum standardized uptake values (maxSUV), and total lesion glycolysis (TLG) as tools for better treatment selection [54].

### 3.2. Pretreatment Organ Function

Multiple studies have shown the importance of several prognostic factors as predictors for organ preservation, treatment response, and outcomes in laryngeal cancer patients [55,56,57]. An area of interest is the pretreatment larynx function and his predictor value for response to RT or chemoradiation. Having a thorough and careful baseline evaluation in a multidisciplinary design approach for the organ function (voice, swallowing) is key in the decision-making process and ultimate patient outcomes. Knowing this crucial information upfront can help to further tailor down treatment options.

### 3.3. Selective Nodal Irradiation

It is known that the risk of lymph node involvement in laryngeal cancer is based on tumor anatomic location, stage, and histology. Historically, patients with locally advanced larynx cancer that require RT as part of their treatment will receive bilateral neck irradiation. A potential way to further de-escalate therapy and decrease morbidity is to perform selective nodal irradiation only in regions at risk of recurrence (>5%), and to lower the elective dose to 40 Gy. The INFIELD phase II trial (Involved Field Elective Volume De-Intensification Radiation Therapy for Head and Neck Cancer) is evaluating this question in oropharyngeal (*n* = 53) and locally advanced laryngeal (*n* = 19) SCC. An IMRT plan with or without chemotherapy is delivered in two sequential courses. The first course delivers 40 Gy in 20 fractions to the gross disease and elective volumes. The second course encompasses a dose of 30 Gy in 15 fractions to the gross disease or 24 Gy in 15 fractions to the microscopic disease and suspicious nodes. Each lymph node is characterized as involved or suspicious, based on anatomic and PET criteria. Level IB will not be electively treated unless it is involved with pathologic or suspicious lymphadenopathy. Level V will not be treated unless two or more ipsilateral nodal levels are involved (or level V itself has pathologic or suspicious adenopathy). Levels III and IV will only be irradiated if the immediately proximal level contains pathologic lymphadenopathy (i.e., level III will be irradiated if level II is positive; level IV will be irradiated if level III is positive). Preliminary data presented at the 2019 annual meeting of the American Society for Radiation Oncology (ASTRO) showed no recurrences in the 40 Gy untreated elective nodal stations after a median follow-up of 11.9 months. This intriguing data requires further validation in a larger setting with longer follow-up [58].

### 3.4. Adaptive Radiotherapy

**Figure 5:**
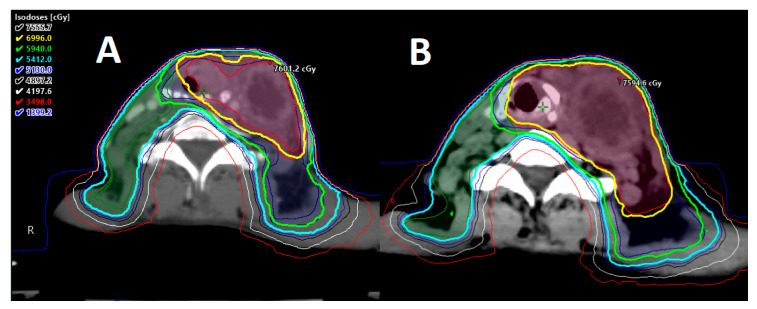
Adaptive radiotherapy. Adaptive planning necessitated by tumor volume changed during the course of radiation. (**A**) Original VMAT treatment plan adapted to accommodate for tumor growth, as depicted by plan in (**B**). Isodoses distribution.

Adaptive RT is the process of re-planning patients during treatment in response to observed spatial and structural changes, e.g., weight loss (anatomy-adaptive RT) and changes in tumor volumes (response-adaptive RT) (Figure 5), or at preset intervals during the treatment course. The use of adaptive RT will allow modifications of the radiation plan based on changes that occur during treatment. In theory, this modality could potentially improve outcomes and reduce toxicity following treatment response. An example is the case of persistent disease, where the use of adaptive RT will allow the radiation oncologist to dose escalate radioresistant disease. Another frequent scenario is the presence of volumetric reductions on tumoral volumes, resulting in unintended dosimetric changes affecting the treatment efficacy and overdosing normal organs like parotid glands, which would ultimately result in increased toxicity. The concept and utility of adaptive RT is promising and continues to evolve [59,60].

### 3.5. Unilateral Neck Irradiation

**Figure 6:**
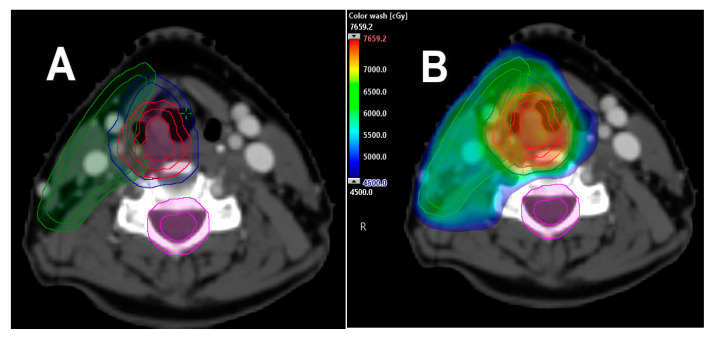
Exemplification of Unilateral neck irradiation case. Unilateral neck irradiation treatment plan for T1N0 squamous cell carcinoma of the right supraglottic larynx, after SPECT/CT with peritumoral 99mTc-nanocolloid injection for lymph drainage mapping. Prescription dose 70 Gy in 35 fractions to high risk PTV. (**A**) Delineation of GTV, high risk CTV/PTV (red), intermediate (blue), and low (green) utilizing 5 mm uniform expansion to render PTV’s. (**B**) VMAT treatment planning technique with partial arcs delivered via simultaneously integrated boost method. Dose color wash distribution.

Due to risks of lymph node involvement in locally advanced laryngeal cancer, patients that require definitive or adjuvant radiation as part of their treatment will receive bilateral neck irradiation. The concept of unilateral neck irradiation has been applied in the last few decades for the treatment of well-lateralized oropharyngeal tumors, with good oncologic and functional results. Lately, with advances in diagnostic imaging and improvements on surgical techniques and radiation delivery, we can envision the possibility of doing unilateral nodal irradiation on well-lateralized laryngeal tumors (Figure 6). Some groups have advocated the use of imaging modalities, such as SPECT/CT, with peritumoral 99mTc-nanocolloid injections for lymph drainage mapping for the planning of unilateral elective nodal irradiation in head and neck SCC. These studies have included patients with well-lateralized T1–3 N0–2b tumors not crossing midline of the oral cavity, oropharynx, larynx, and hypopharynx. Lymphatic drainage was successfully visualized in 98% of patients. Twenty percent of patients had visible contralateral drainage in levels II (88%), III (25%), and IV (13%), with an observed increased risk of contralateral drainage associated with higher tumor stage (T3 (45%) vs. T1–T2 (14%) tumors) [61]. Two comparison radiation plans (standard bilateral neck vs. selective SPECT/CT-guided ipsilateral neck irradiation) were created for each case. Radiation doses to organs at risk were evaluated, and the clinical benefits were predicted using different normal tissue complication probability (NTCP) models [62]. Using this approach, a total of 50 patients were treated. With a median follow-up of 33 months, only one patient (2%) had contralateral regional failure. SPECT-guided elective nodal irradiation was associated with lower rates of dysphagia, PEG tube placement, and late xerostomia compared to standard bilateral nodal irradiation [63].

This concept can also be translated into patients treated with surgery upfront. The current clinical trial NCT03622164 is evaluating the role of unilateral neck RT in patients with squamous cell carcinomas of the head and neck (oral cavity, oropharynx, larynx, or hypopharynx) undergoing primary surgical resection and bilateral, modified radical, or selective neck dissections, with ≥10 pathologically negative lymph nodes removed on the contralateral neck that will require adjuvant RT, based on final pathologic features.

If unilateral neck irradiation is perhaps indicated, treatment with charged particles, such as proton therapy using modern delivery techniques, like intensity modulated proton therapy (IMPT) [64] or individual field simultaneous optimization (IFSO) [65], could further spare organs at risk and the contralateral neck, due to the physical properties of charged particles.

### 3.6. Omission of Resected Neck—Radiation to the Primary Surgical Bed Only

**Figure 7:**
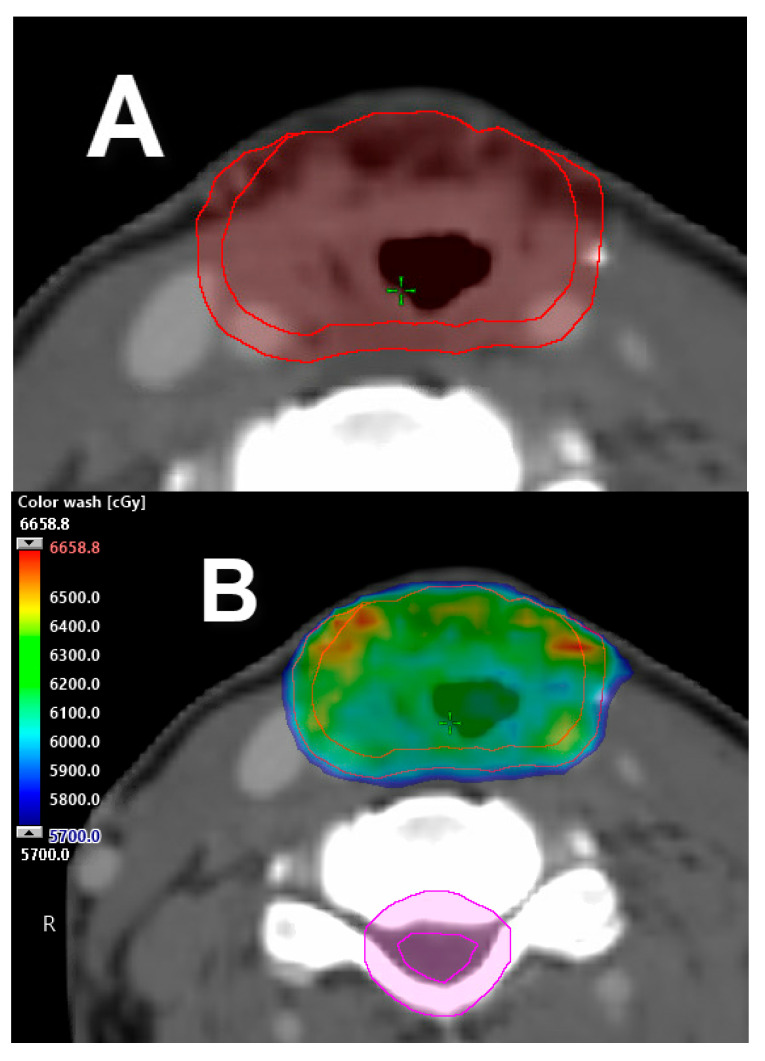
Exemplification treatment plan of omission of resected neck – radiation to the primary surgical bed only. (**A**) CTV (red) contents include the primary post-operative bed for pT3 N0 squamous cell carcinoma of glottic larynx with close margins, positive lymphovascular and perineural invasion, with >90 lymph nodes negative in the bilateral cervical neck(s). PTV is rendered using uniform 5 mm expansion from the CTV (red), cropped back 3 mm from the skin’s surface for build-up. (**B**) VMAT treatment planning technique for the primary, post-operative surgical bed only, depicted with a total prescription of 60 Gy delivered in 30 fractions to PTV. Dose color wash distribution.

A phase II trial from Washington University omitted postoperative radiation to the pathologically node-negative neck in patients with primary head and neck squamous cell carcinoma. Seventy-two patients with tumors of the oral cavity, oropharynx, larynx, and hypopharynx were included. After oncologic resection (primary and neck dissection), patients with a pathologically negative neck (pN0), and with risk features mandating adjuvant radiation, were treated only at the primary tumor bed. The median of sampled lymph nodes on the ipsilateral neck was 28.5. For the contralateral neck dissection, 10 or more lymph nodes were resected in 88% of patients. At a median follow-up of 53 months, only two patients were documented with recurrences on the pathologically negative and unirradiated neck, for a regional control of 97%. Of note, the two patients with neck failures also experienced local recurrences [66] (Figure 7).

### 3.7. Radiation to Neck(s) Only

**Figure 8:**
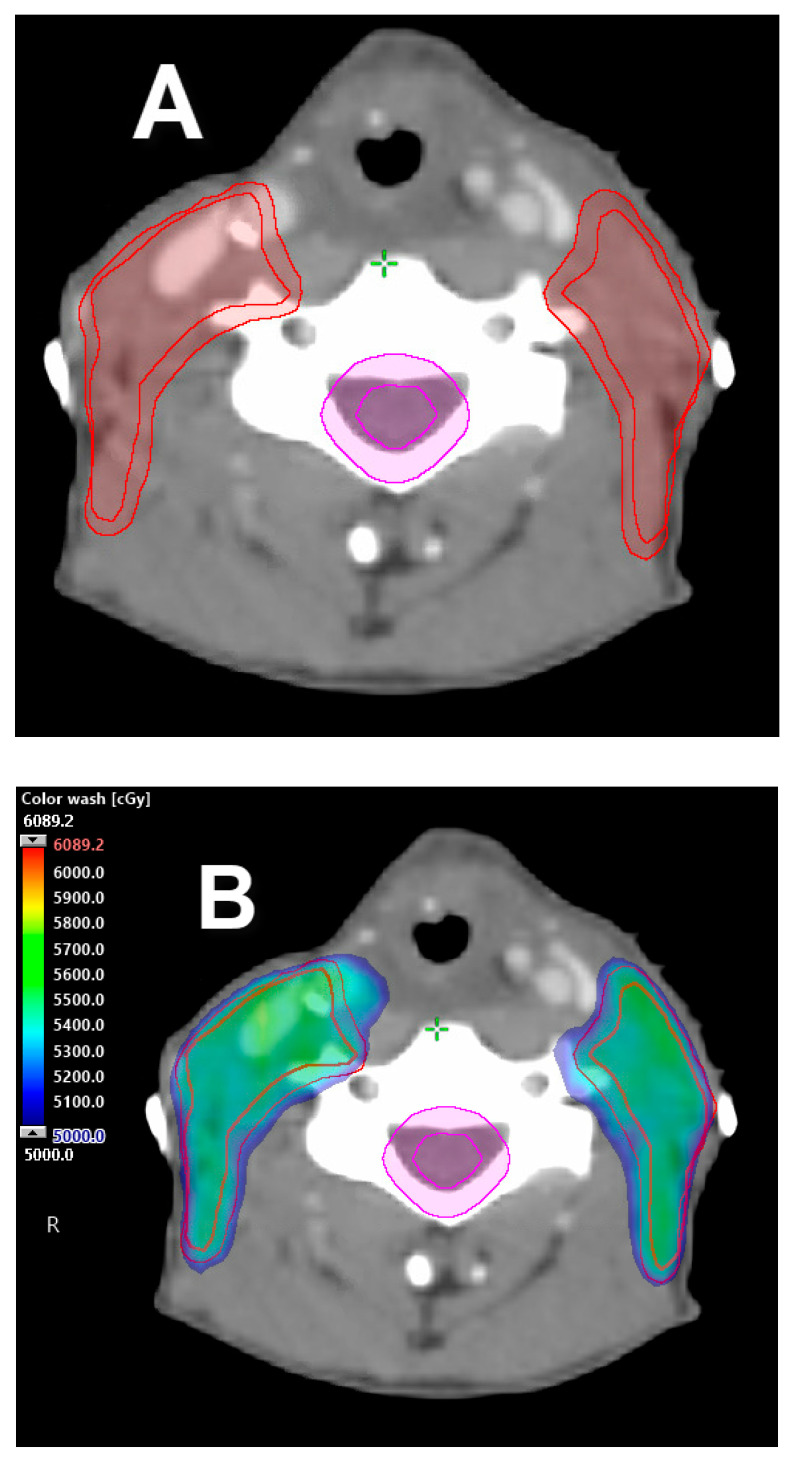
Exemplification treatment plan of radiation to neck(s) only. (**A**) Bilateral neck CTV (red) shown with 5 mm uniform expansion to render PTV. PTV is cropped 3 mm from skin surface for build-up. (**B**) VMAT treatment plan to the bilateral neck(s) avoids the primary surgical bed in a resected pT3 N2 SCC of the glottic larynx showing no adverse pathologic risk features in the primary tumor specimen/surgical bed, with indications for adjuvant radiation as a result of multiple positive lymph nodes. Prescription dose 54 Gy in 27 fractions. Dose color wash distribution.

Similar to the concept of sparing the pathologically negative neck, it may be possible to omit the primary surgical bed in the radiation field in the circumstance of a resected primary tumor that shows no adverse risk features on pathology, such as positive/close margins, perineural invasion (PNI), lymphovascular invasion (LVI), or indications for adjuvant radiation based only on pathologic analysis of the neck (number of positive lymph nodes or extranodal extension (ENE)) (Figure 8). This treatment approach is currently being evaluated in other anatomic sites of the head and neck, such as the oropharynx [67,68,69].

### 3.8. Nanoparticle Therapy

One area of increased research interest in the treatment of head and neck cancers is nanoparticle therapy. Theoretically nanoparticles can be used as vectors to target the tumor site by achieving controlled drug release, or to selectively potentiate radiation dose deposits while keeping toxicity lower in the surrounding normal tissues. Preclinical studies using nanoparticles as radiosensitizers have shown an improved therapeutic index with increased tumor control efficacy and safety toxicity profile. Their potential clinical applications are currently under investigation [70]. NBTXR3 is an aqueous suspension of crystalline hafnium oxide that, after intramural injection prior to the first radiation fraction, increases the energy deposited from ionizing radiation within the cancer cell without increasing doses to the surrounding normal tissues. Preliminary data of a phase I/II trial of NBTXR3 nanoparticles activated by IMRT in the treatment of local advanced-stage SCCs of the head and neck in elderly or frail patients who are ineligible for cisplatin or intolerant to cetuximab showed the efficacy of the radioenhancer [71]. Coming up is NBTXR3-312, a phase III trial for elderly patients with locally advanced disease ineligible for cisplatin therapy. Other possible clinical applications are nanoparticle technology in combination with anti-PD1 inhibitors (NCT03589339).

### 3.9. Deep Machine Learning: Radiomics

The use of machine learning algorithms in the field of artificial intelligence has the potential to help identify and evaluate pretreatment tumor characteristics that could be considered for physician decision-making. The spectrum of machine learning applications is extensive, promising, and continues to evolve. Some applications in the area of radiation planning are automatic organ-at-risk delineation and adaptive radiotherapy. Machine learning may also be useful in identifying and developing normal tissue complication probability (NTCP) models [72]. Kann et al. [73] used deep-learning algorithms to identify pretreatment extranodal extensions (ENEs) in the SCCs of the head and neck, and compared their own performance against two board-certified neuroradiologists. Preoperative, contrast-enhanced CT scans and pathology results from two external data sets were employed for analysis. The computer algorithm was able to achieve an area under the receiver operating characteristic curve (AUC) of 0.84, outperforming the neuroradiologists’ AUCs of 0.70 and 0.71. Detection of ENE prior to treatment can help with clinical decision-making, since most patients with ENEs in the final pathologic specimen will require additional therapy. Using machine deep-learning assistance may improve the accuracy of diagnostic radiologists.

Another potential advantage of the use of deep machine learning is the evaluation of anatomic and functional changes during treatment to predict toxicity and improve functional outcomes. Scalco et al. [74,75] applied texture analysis to CT images obtained during RT to evaluate and monitor structural and anatomic changes of the parotid glands, and whether these changes could characterize preclinical signs of xerostomia.

Similarly, the Johns Hopkins group explored radiomorphologic dose patterns in salivary glands (parotid and submandibular glands) that could predict xerostomia three months after treatment. The ridge logistic regression method was used to evaluate the influence patterns of doses in the salivary glands on xerostomia. It was found that the superior–anterior portion of the contralateral parotid gland and the medial portion of the ipsilateral parotid gland were the most influential areas regarding dose effect on xerostomia [76]. Furthermore, the spatial radiation dose influence on the recovery of xerostomia eighteen months after treatment was also analyzed. This analysis found that the superior portions of the two parotid glands are the most influential in xerostomia recovery [77]. Machine learning methods have the potential to help optimize and improve radiation treatment planning and decrease toxicity after treatment.

Radiomics is a method that works through characterization algorithms to extract and analyze large amounts of dimensional data from radiographic medical images, helping to personalize treatment. For example, radiomics can identify regions of radioresistant tumoral cells and help the radiation oncologist to selectively intensify or escalate radiation dose to those areas [78]. It can also provide information about tumor heterogeneity/segmentation and predict pathological grading, gene expression profiling, oncologic outcomes, and risk stratification, as well as monitoring tumor response and normal tissue changes after RT [79,80].

### 3.10. Tumor Heterogeneity

It is well-established that molecular variations between (intertumor heterogeneity) or within tumors (intratumor heterogeneity) exist. Tumor heterogeneity contributes to the ambiguous clinical responses and outcomes observed in clinical practice. For example, it is believed that therapeutic resistance may develop as a result of treatment-induced evolution and natural selection of cancer sub-clones. Tissue and tumor analysis and profiling will further help to aid in the understand of molecular changes and mechanisms implicated. Furthermore, improved understanding of tumor heterogeneity may also assist in the development of biomarkers and selection/stratification of patients into distinctive prognostic groups, allowing patients to receive precision cancer medicine [81].

### 3.11. MRI-Guided Radiotherapy

The use of MRI in laryngeal cancer can be useful to better evaluate tumor extension/invasion into the pre- and paraglottic space, cricoarytenoid unit, and subglottic and base of tongue regions in locally advanced tumors. It can also be helpful to assess perineural tumor spread and vascular involvement. Due to its superior soft-tissue contrast resolution, MRI can be particularly advantageous for a more precise target volume delineation, and additionally reduce or adapt treatment volumes. Moreover, with recent advances in the delivery of imaged-guided RT, we can envision the use of real-time, MRI-guided RT to track anatomical motion during treatment, and account for respiratory and deglutition real-time motion of the larynx to assist in more accurate treatment delivery, allowing further reduction in treated volumes, and improving the therapeutic ratio [82,83,84,85,86].

### 3.12. Ultra-High-Dose-Rate (FLASH) Radiotherapy

Conventional head and neck RT is delivered in protracted courses of 6–7 weeks, with daily dose fractions of 1.8–2.0 Gy (standard fractionation) at dose rates around 0.01–0.03 Gy/s. The total dose delivered is determined by tumor type, location, and dose tolerance limits of surrounding normal tissues. Recently, with the development of newer technology, it is possible to deliver highly curative radiation doses (≥10 Gy) to tumors at ultra-high dose rates (≥40 Gy/s), known as “FLASH” irradiation. Preclinical studies in animals have shown relative protection of normal tissues, perhaps due to the very short time of exposure, while maintaining the antitumor response of conventional radiation [87,88,89]. The mechanism of the underlying effects observed in FLASH RT and its clinical applications remains under investigation [90].

### 3.13. Radiotherapy Coupled with Biological Agents

The combined effects of ionizing radiation and biological agents have gained considerable interest in the recent past. Different agents, including monoclonal antibodies (cetuximab, bevacizumab) [91], proteasome inhibitors (bortezomib) [92], immunomodulatory drugs (nivolumab, pembrolizumab) [93,94], intra-tumoral TNFerade [95], Aurora kinase A (AURKA) and WEE1 inhibitor [96] [NC03028766], inhibitors of the enzyme poly-ADP ribose polymerase (PARP inhibitor, i.e., olaparib) [97] [NCT02229656], and peptidomimetics of the second mitochondrial-derived activator of caspases (SMAC mimetic, i.e., birinapant) [98] [NCT03803774] have or are currently being studied to evaluate their synergistic effects with RT. The current data of combining radiation and biological agents is mixed, both in clinical response and also in toxicity profiles.

## 4. Future Directions

The current management of laryngeal cancer is standardized based on tumor extent/stage, location, histology, organ function, and patient preference. Continued research, translation, and incorporation of new promising approaches into clinical practice will help evolve the process of laryngeal cancer treatment to become more personalized.

## 5. Conclusions

The treatment of larynx cancer remains challenging. A multi-disciplinary approach is key for treatment success. There are multiple, promising novel strategies to better select and individualize the treatment of laryngeal cancer. Although most of these concepts remain under investigation, the presented data in this review suggest their potential to continue to improve the therapeutic ratio and quality of life of these patients. Newer studies incorporating these ideas in the clinical setting in a prospective manner with a larger number of patients are warranted, in order to further determine their safety and efficacy.
